# A Panel of Five-lncRNA Signature as a Potential Biomarker for Predicting Survival in Gastric and Thoracic Cancers

**DOI:** 10.3389/fgene.2021.666155

**Published:** 2021-04-13

**Authors:** Jiayue Shao, Wei Lyu, Jiehao Zhou, Wenhui Xu, Dandan Wang, Shanshan Liang, Jiayin Zhao, Yujing Qin

**Affiliations:** ^1^Department of Medical Oncology, Cancer Hospital, Harbin Medical University, Harbin, China; ^2^Department of Pathology, Guangdong Women and Children Hospital, Guangzhou, China; ^3^Department of Gastroenterology, Heilongjiang Provincial Hospital, Harbin, China; ^4^Department of Gastroenterology, Heilongjiang Provincial Hospital, Harbin Institute of Technology, Harbin, China

**Keywords:** long non-coding RNA, gastric cancer, overall survival, thoracic malignancy, prognosis

## Abstract

Dysfunctional long non-coding RNAs (lncRNAs) have been found to have carcinogenic and/or tumor inhibitory effects in the development and progression of cancer, suggesting their potential as new independent biomarkers for cancer diagnosis and prognosis. The exploration of the relationship between lncRNAs and the overall survival (OS) of different cancers opens up new prospects for tumor diagnosis and treatment. In this study, we established a five-lncRNA signature and explored its prognostic efficiency in gastric cancer (GC) and several thoracic malignancies, including breast invasive carcinoma (BRCA), esophageal carcinoma, lung adenocarcinoma, lung squamous cell carcinoma (LUSC), and thymoma (THYM). Cox regression analysis and lasso regression were used to evaluate the relationship between lncRNA expression and survival in different cancer datasets from GEO and TCGA. Kaplan-Meier survival curves indicated that risk scores characterized by a five-lncRNA signature were significantly associated with the OS of GC, BRCA, LUSC, and THYM patients. Functional enrichment analysis showed that these five lncRNAs are involved in known biological pathways related to cancer pathology. In conclusion, the five-lncRNA signature can be used as a prognostic marker to promote the diagnosis and treatment of GC and thymic malignancies.

## Introduction

Long non-coding RNAs (lncRNAs) play an important regulatory role at the transcriptional, post-transcriptional, and epigenetic levels and also participate in multiple aspects of gene regulation and a variety of biological processes (Kopp and Mendell, 2018). As a type of non-coding RNA, lncRNA, which has no protein-coding ability and is more than 200 bp in length, can be transcribed by RNA polymerase II. Abnormal lncRNA expression has also been observed in many complex human diseases, especially cancer. Cancer-related lncRNAs show abnormal expression patterns in a tissue-or cancer-specific manner, indicating their potential as new independent biomarkers for cancer diagnosis and prognosis (Wang et al., 2015; Tate et al., 2019). For example, the lncRNA MALAT1 can regulate multiple biological processes during the development of several cancers. The diagnostic and prognostic significance of MALAT1 has been demonstrated in gliomas and different cancers of the breast, lung, ovary, pancreas, and prostate (Goyal et al., 2021). The overexpression of the lncRNA GHET1 predicts unfavorable survival and clinical parameters in patients with various cancers (Jiang et al., 2019).

Gastric cancer (GC) is the fourth most common malignant tumor and the third leading cause of cancer-related death (Siegel et al., 2021). Although the treatment of GC has made great progress in recent years, the prognosis remains poor, with a 5 years survival rate of less than 40% (Allemani et al., 2018). In addition, the pathology of several thoracic malignancies, including lung cancer, esophageal cancer, and breast cancer, is found to exhibit dysregulated lncRNA expression in a variety of malignancies. Traditional classification methods are not effective, and molecular biological markers can reveal tumor progression and survival in cancer patients. Thus, identifying specific lncRNAs to predict prognosis is very important for improving patient survival.

To establish a novel risk-stratification lncRNA signature panel in different cancers, we collected lncRNA expression data and clinical information of GC and several thoracic malignancies, such as breast invasive carcinoma (BRCA), esophageal carcinoma (ESCA), lung adenocarcinoma (LUAD), lung squamous cell carcinoma (LUSC), and thymoma (THYM) patients from different independent databases to identify and develop a novel lncRNA-based signature panel as an independent predictor of cancer prognosis. Univariate and multivariate Cox regression analyses were used to explore the prognostic efficiency of lncRNAs. Five potential lncRNA biomarkers significantly related to the survival of GC patients were identified, and a risk score model was established using the coefficients of multivariate Cox regression analysis to predict the survival of patients. Furthermore, this model was applied to several thoracic malignancies in the TCGA database. We found that this five-lncRNA signature could also divide patients into different risk groups in BRCA, LUSC, and THYM. Furthermore, the possible molecular mechanisms related to the occurrence and progression of these prognostic lncRNAs were investigated using functional analysis. In conclusion, the five-lncRNA signature panel offers a potential platform for risk stratification in patients with GC and several thoracic malignancies, which has great implications in the clinical management of patients and contributes to disease diagnosis.

## Materials and Methods

### Data Collection

The gene expression profiles and corresponding clinical information of GC and several thoracic cancers used in this study were obtained from the publicly available Gene Expression Omnibus (Barrett et al., 2013) (GEO)^1^ database and The Cancer Genome Atlas (Tomczak et al., 2015) (TCGA)^2^ database. GSE62254 (*n* = 300) and GSE15459 (*n* = 192) datasets were collected from GEO. The datasets of BRCA (*n* = 1,089), ESCA (*n* = 163), LUAD (*n* = 515), LUSC (*n* = 497), and THYM (*n* = 119) were collected from TCGA.

### Identification of Potential lncRNA Biomarkers

The GSE62254 series samples were randomly generated in a training set and a test set. Univariate Cox regression analysis was performed on the training set to evaluate the correlation between the expression level of each lncRNA and patient overall survival (OS) (Guo et al., 2019). Lasso regression (Xu et al., 2018; McEligot et al., 2020), which solves the over-fitting problem and can directly reduce some repetitive unnecessary parameters to zero in the parameter reduction process, was used to further screen the results from univariate Cox regression. The results of lasso algorithm screening were used to construct a multivariate Cox risk regression model, where OS was the dependent variable and the other clinical information was the covariate. We retained the lncRNA (*p* < 0.01) as the final biomarker, which is significantly related to the OS of GC patients. When using the Cox risk regression model, the PH hypothesis test (Zhang et al., 2018) was also used to test whether the data conformed to the Cox regression model. To verify the reliability of the risk score model, we used the test set as well as two other sets of data for validation.

### Statistical Analysis

The Kaplan-Meier survival curve (Ranstam and Cook, 2017) was used to prove the difference in OS between the high-risk and low-risk groups, and a bilateral logarithmic rank test was used to evaluate whether the difference was statistically significant (Guyot et al., 2012). Univariate and multivariate Cox regression analyses were performed for each dataset, with OS as the dependent variable and lncRNA risk score as explanatory variables. All analyses were conducted using R (v3.4.1) software.

### Functional Enrichment Analysis

Pearson correlation analysis was used to evaluate the co-expression relationship of biomarker lncRNAs related to the OS of GC patients and mRNA. We used the cor.test function in R to calculate the correlation coefficient and significance level between the lncRNA and protein-coding genes. We defined the significant correlation level as a *p* < 0.05 and ranked the positive and negative correlation coefficients. The top 100 protein-coding genes were used for subsequent functional analysis. Functional enrichment of the R package clusterProfiler (Yu et al., 2012) was used to analyze the functional enrichment of mRNAs co-expressed with biomarker lncRNAs to predict the biological function of these biomarkers (Zhou et al., 2019).

### Combination of Biomarkers From Gastric Cancer and Prognosis of Thoracic Malignancies

The RNA-seq profiles of thoracic malignancies, including BRCA, ESCA, LUAD, LUSC, and THYM, were downloaded from TCGA. Furthermore, we used a univariate Cox risk regression model to explore the association between biomarkers discovered in GC and other thoracic malignancies.

## Results

### Five lncRNAs Identified to Be Associated With GC Prognosis

**FIGURE 1:**
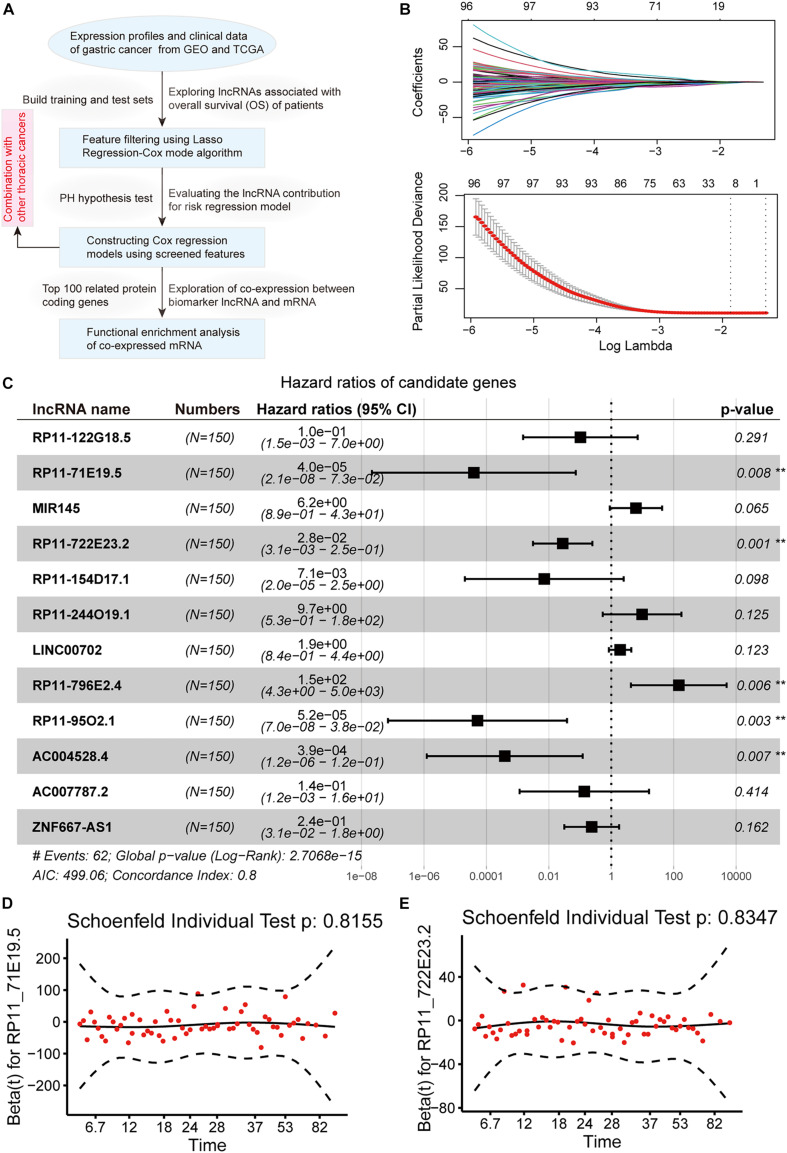
Identification of lncRNAs associating with gastric cancer prognosis. **(A)** The workflow of our analysis pipeline. **(B)** Lasso regression model screened characteristic lncRNAs. Variation curve of regression coefficient and β value. **(C)** Forest plot of multivariate Cox regression results, which include *p-*value and confidence interval of hazard ratios. **(D,E)** The diagram shows the results of the PH hypothesis test. The horizontal axis represents the survival time, and the vertical axis represents the Schoenfeld residual.

To explore the lncRNAs related to the OS of GC patients, microarray profiles from GSE62254, which have appropriate sample abundance and clinical data, were used to construct regression models. The workflow of the analysis pipeline is shown in Figure 1A. For the GSE62254 series, we randomly divided samples into a training set (*n* = 150) and a test set (*n* = 150) with the same amount. In the training set, the expression profile of lncRNA was selected for analysis by univariate Cox regression analysis with OS, including survival time and status as the dependent variable. We found 580 lncRNAs (*p* < 0.05 and PH inspection *p* > 0.5) whose expression levels were significantly related to patient survival. Considering the large abundance of the initially screened features, lasso regression was used for a more rigorous screening (Figure 1B). We identified 12 significant candidate markers for lncRNA. Furthermore, we used the selected few candidate lncRNAs to establish a multivariate Cox regression model. When considering the interaction between lncRNAs and clinical features, only five lncRNAs (RP11_71E19.5, RP11_722E23.2, RP11_796E2.4, RP11_95O2.1, and AC004528.4) were significantly associated with patient survival (Figure 1C). At the same time, the PH hypothesis test results showed that there was no obvious correlation between the Schoenfeld residual and the rank of survival time (Figures 1D,E). We used these five biomarker lncRNAs to construct a risk score model independent of other clinical features.

### Evaluating the lncRNA Contribution for Risk Prediction Model

**FIGURE 2:**
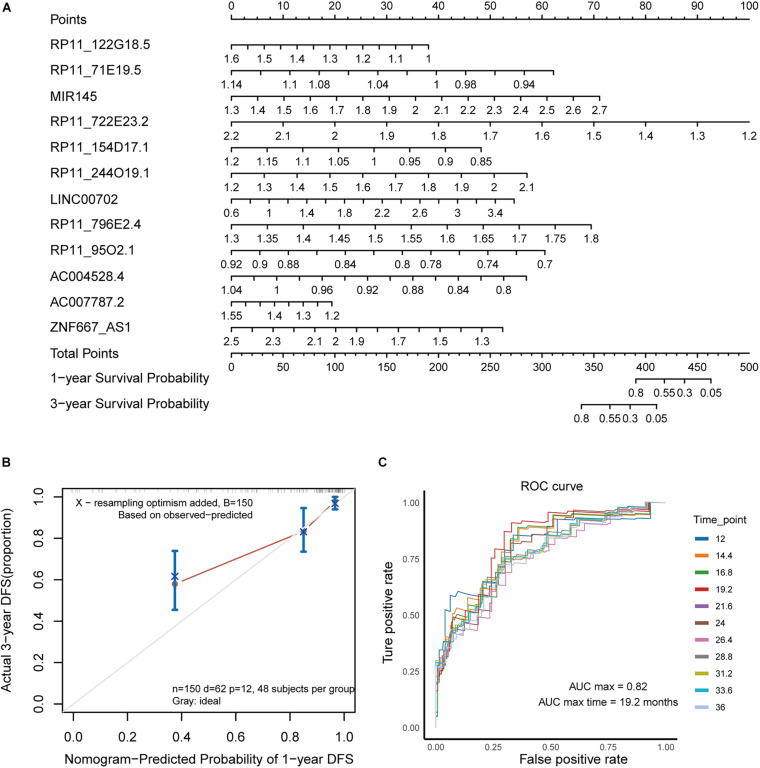
Evaluating the lncRNA contribution for risk prediction model. **(A)** The nomogram shows the prediction model of survival probability. **(B)** Calibration curve of the nomogram. **(C)** The ROC curve reflects the performance of the Cox risk regression model in predicting the probability of survival of patients at different time nodes.

For the multivariate Cox risk prediction results, we used the nomogram method to build a more intuitive prediction model of 1 and 3 years survival time probabilities. We found that the characteristic lncRNAs that are more relevant to the patient’s survival will increase in scores under the same gene expression fluctuations (Figure 2A). To evaluate the contribution of the prediction model, we used a calibration curve to show the predicted survival probability of the prediction model for 1 and 3 years. We found that the fluctuation of the fitting results was not obvious, suggesting that the risk regression model we built was reasonable (Figure 2B). A receiver operating characteristic curve was also computed to evaluate the sensitivity and specificity of the predictive model. We found that the AUC value of the prediction model at different time points was approximately 0.8, and it had the best predictive ability (AUC = 0.82) at 19.2 months (Figure 2C). Taken together, these results suggest that the risk prediction model built with five biomarker lncRNAs has a good contribution to the prediction of the survival time of GC patients.

### Construction of a Risk Score Model for GC

**FIGURE 3:**
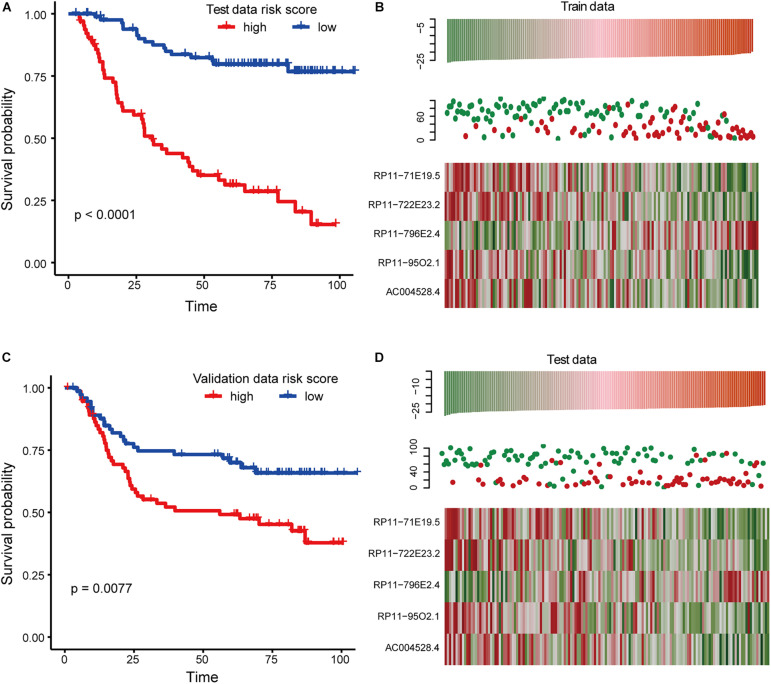
Survival analysis in training and testing dataset. **(A)** Kaplan-Meier curve reflects the difference of overall survival (OS) between high and low-risk score samples for the training set. **(B)** This graph reflects the expression levels of biomarkers for the training set as well as the survival status and risk scores of the patients. **(C)** The same as in **(A)** but for the test set. **(D)** The same as in **(B)** but for the test set.

Using the coefficients obtained from the multivariate Cox regression analysis, we constructed a risk scoring model (Wang et al., 2019; Guo et al., 2020) to predict the patient’s OS, as shown below: risk score = (−10.14 ^∗^ expression value of RP11-71E19.5) + (−3.59 ^∗^ expression value of RP11-722E23.2) + (4.98 ^∗^ expression value of RP11-796E2.4) + (−9.868541 ^∗^ expression value of RP11-95O2.1) + (−7.857590 ^∗^ expression value of AC004528.4). Based on this model, a risk score was calculated for each patient in the training set, and patients were divided into a high-risk group (*n* = 75) and a low-risk group (*n* = 75) using the median risk score as the cut-off point. We found that there was a significant difference in the OS between the high-risk group and the low-risk group, and the *p*-value of the logarithmic rank test was less than 0.0001 in the Kaplan-Meier curve (Figure 3A). Univariate Cox regression analysis also revealed that risk scores constructed from five biomarker lncRNAs were significantly associated with OS (*p* < 0.01). We also evaluated the relationship between the risk score and the distribution of lncRNA expression in the training set. The expression of RP11-796E2.4 in the high-risk score tended to be upregulated, while the expression of the remaining four lncRNAs tended to be downregulated (Figure 3B). We also applied a risk score model to calculate the risk score of each sample in the test dataset. Similar to the result predicted by the risk score obtained in the training set, the test data set also found that patients with high-risk scores had significantly worse OS than patients with low-risk scores (Figures 3C,D).

### Validating Risk Score Models in Multiple Expression Profiles

**FIGURE 4:**
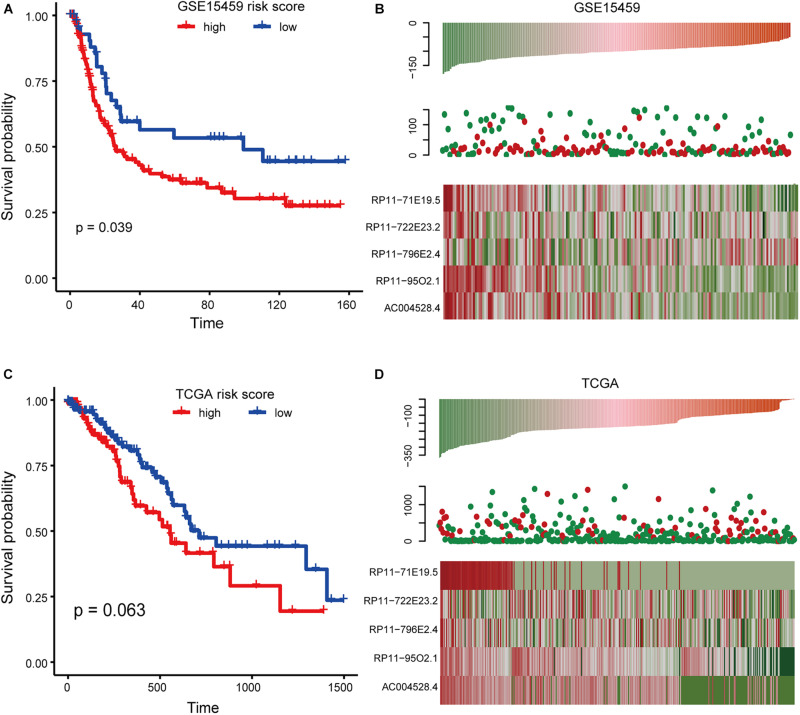
Survival analysis in the GSE15459 and TCGA dataset. **(A)** Kaplan-Meier curve of risk score model based on GSE15459 data. **(B)** Expression pattern of lncRNA and survival status and risk score of patients based onGSE15459 data. **(C)** The same as in **(A)** but for the RNA-seq profile from TCGA. **(D)** The same as in **(B)** but for the RNA-seq profile from TCGA.

To further investigate the robustness and practical application of the five-lncRNA risk scoring model, GSE15459 and RNA-seq profiles of GC from TCGA were also used to validate the risk scoring model. We divided the samples from the GSE15459 series into the high-risk group (*n* = 96) and the low-risk group (*n* = 96). There was a significant difference in the OS between the two groups (Figure 4A). Univariate Cox regression analysis also showed that the risk score was significantly correlated with OS (*p* = 0.0309, *HR* = 1.000187). We found that the risk score distribution, survival status, and lncRNA expression of all patients were consistent with those observed in the training dataset (Figure 4B). In the TCGA dataset, 380 patients in the TCGA data set were divided into a high-risk group (*n* = 190) and a low-risk group (*n* = 190). The results of univariate Cox regression analysis also showed that the risk score was significantly correlated with OS (Figures 4C,D). All these results indicate that the risk score model has a better universality.

### Cancer-Promoting Function of mRNA Co-expressed With Five lncRNAs

**FIGURE 5:**
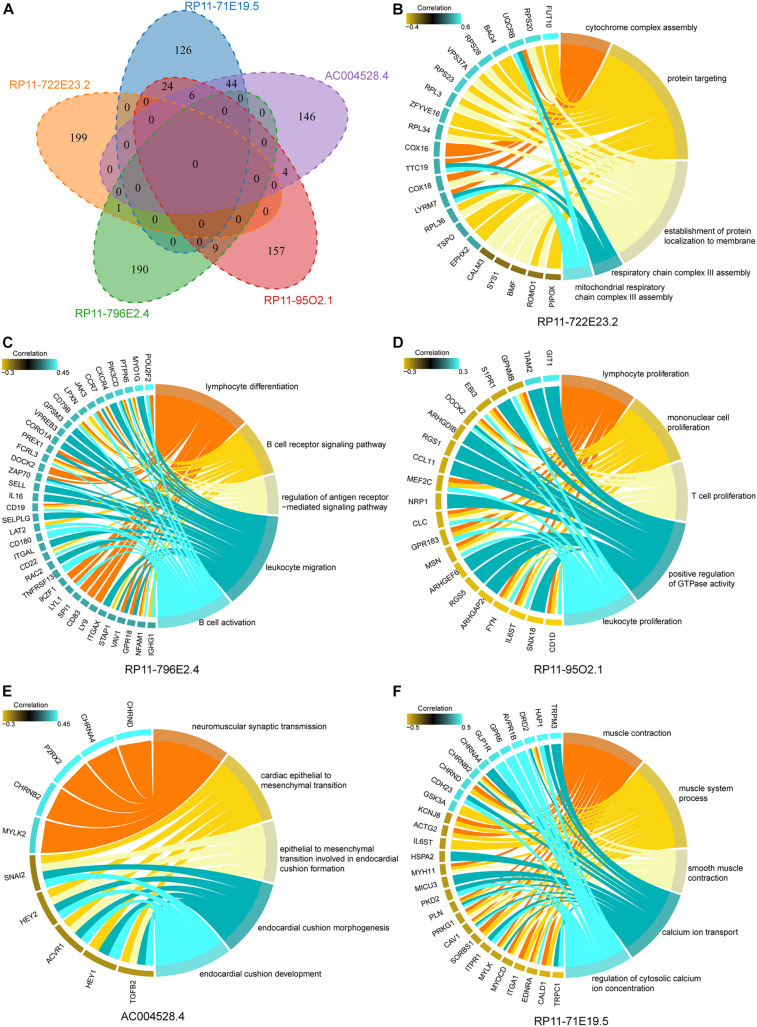
Cancer-promoting function of mRNA co-expressed with five lncRNAs. **(A)** The Venn diagram shows the intersection of mRNAs related to the expression of five biomarker lncRNAs. **(B–F)** The results of functional enrichment of mRNA relating to the expression of lncRNAs. Protein coding genes are on the left side of the circle diagram, and biological pathways (BP) are on the right.

To explore the functional significance of five lncRNA biomarkers in the occurrence and development of gastric carcinoma, bioinformatics analysis was carried out to predict the function of lncRNAs. We extracted these five lncRNA expression profiles and all mRNA expression profiles from the GSE62254 dataset and calculated the Pearson correlation coefficient between each pair of lncRNAs and mRNAs. We found that the mRNAs related to these five lncRNAs had no significant intersection (Figure 5A), indicating that the five lncRNAs have independent roles in carcinogenic mechanisms.

Functional enrichment analysis was performed to reveal the functional mechanism of biomarker lncRNAs. We found many biological pathways (BPs) influenced by mRNAs that correlate with biomarker lncRNA. For lncRNA RP11-722E23.2, the function was mainly enriched in energy metabolism (Figure 5B). Variation in lncRNA RP11-796E2.4 and RP11-95O2.1 expression mainly affects immune regulation, which is essential for the recognition and elimination of gastric tumor cells (Figures 5C,D; Chen et al., 2007; Amedei et al., 2012). AC004528.4 was mainly involved in the epithelial-mesenchymal transition, which regulates the invasion and metastasis of cancer cells (Lamouille et al., 2014; Dongre and Weinberg, 2019; Figure 5E). RP11-71E19.5 mainly regulated the ion channels and contractile activity of gastric muscle cells (Figure 5F). These results indicate that lncRNAs can promote the occurrence and development of GC through multiple mechanisms.

### Exploring the Prognostic Efficiency of Five lncRNAs in Thoracic Malignancies

**FIGURE 6:**
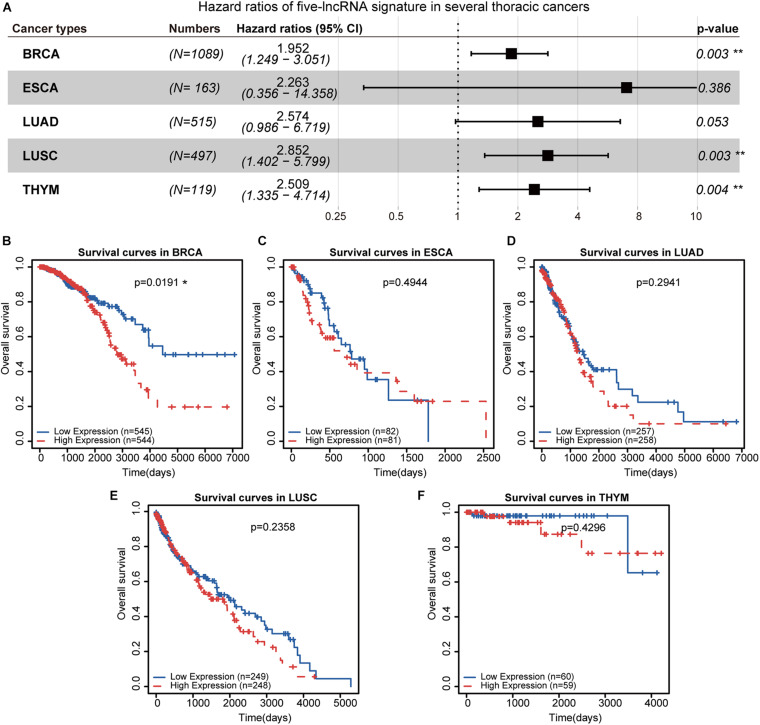
Survival analysis in several thoracic cancers. **(A)** The forest plot demonstrates the risk ratios of five prognosis-related lncRNAs in thoracic cancer. **(B–F)** Kaplan-Meier curves of the five prognostic lncRNAs in thoracic cancers. Patients were divided by the median risk score. **p < 0.01.

Furthermore, we evaluated the prognostic performance of these five-lncRNA signatures in several thoracic cancers, including BRCA, ESCA, LUAD, LUSC, and THYM. The five-lncRNA signature was found to be significantly associated with patient survival in BRCA (*HR* = 1.952, 95% CI = 1.249–3.051, *p* = 0.003), LUSC (*HR* = 2.852, 95% CI = 1.402–5.799, *p* = 0.003), and THYM (*HR* = 2.509, 95% CI = 1.335–4.714, *p* = 0.004) (Figure 6A). Patients with different thoracic cancers were ranked and divided into two risk groups based on the median risk score. Kaplan-Meier survival analysis was performed to evaluate the prognostic significance between the two risk groups in each dataset (Figures 6B–F). We found that the OS of BRCA patients was significantly different between the high- and low-risk groups (Figure 6B). These results were consistent with previous studies that reported that breast cancer with metastasis to the gastric tissue was often mistaken for primary GC (Kim et al., 2018; Birla et al., 2019). However, the survival of patients with other thoracic malignancies did not show sensitivity to these five lncRNAs in GC (Figures 6C–F).

**FIGURE 7:**
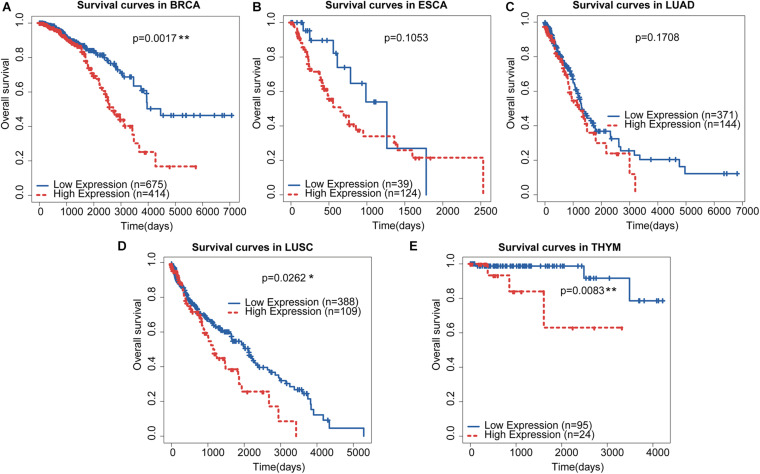
Re-dividing of thoracic cancer patients and survival analysis. **(A–E)** Kaplan-Meier curves of the five prognostic lncRNAs in thoracic cancers. Patients were divided by the optimal cut-off identified from the minimum *p-*value approach.

Using the median score as a threshold may arbitrarily divide patients with continuous gene expression into different groups and fail to obtain significant survival results. We employed the minimum *p*-value approach for grouping patients and determined the optimal cut-off point for continuous gene expression measurement (Mizuno et al., 2009). Based on this strategy, we re-divided the patients with these thoracic cancers into different groups according to the minimum *p*-value (Figures 7A–E). We found that the OS of BRCA (Figure 7A), LUSC (Figure 7D), and THYM (Figure 7E) were significantly different in the high-and low-risk groups, which was consistent with the Cox regression analysis results in Figure 6A. Previous studies have also shown that advanced LUSC is often accompanied by gastric metastasis (Li et al., 2018; He et al., 2019). All these results suggest that the similarity in molecular mechanisms between GC and BRCA and LUSC may be caused by a similar localized pathological tissue origin. Taken together, the five-lncRNA signature identified in GC may be a potential prognostic marker for several thoracic malignancies, including BRCA, LUSC, and THYM, which will provide new avenues for the diagnosis and treatment of these cancers.

## Discussion

In this study, we identified five lncRNAs related to patient survival in gastric carcinoma and established a risk score model. The reliability of the model was verified using data from other public GCs. Furthermore, these five lncRNAs were also associated with the OS of BRCA, LUSC, and THYM patients. To some extent, the model has superior performance compared to known traditional clinical parameters. These results demonstrate the potential application of lncRNA as a new independent biomarker for the diagnosis and prognosis of GC.

As one of the common malignant tumors, GC has a high mortality rate (Hartgrink et al., 2009; Smyth et al., 2020). Although treatment has made great progress, the prognosis remains poor. LncRNAs play an important regulatory role at the transcriptional, post-transcriptional, and epigenetic levels and participate in multiple aspects of gene regulation and a variety of biological processes (Batista and Chang, 2013). In recent years, lncRNAs have been found to play an important role in cancer. Therefore, our exploration of biomarkers that affect tumor progression and survival of GC patients is of great significance for the diagnosis and treatment of GC.

We collected several expression profiles and the corresponding clinical information of GC, including GSE62254, GSE15459, and RNA-seq profiles from TCGA. The GSE62254 data were randomly divided into training and test sets. We used the lasso regression-Cox mode algorithm to screen for features related to the patient’s OS. Compared with other feature filtering algorithms such as linear regression and ridge regression, the lasso algorithm solves the overfitting problem and can directly reduce some repetitive and unnecessary parameters to zero in the parameter reduction process. Lasso regression performs well in controlling the number of features (Xu et al., 2018; McEligot et al., 2020). Further, multivariate Cox regression analysis identified five lncRNAs that could independently predict the OS of patients. Subsequently, a risk-scoring model was established using the coefficients of multivariate Cox regression analysis. The training data set was divided into high-risk and low-risk groups according to the median risk score, and there was a significant difference in OS between the two groups. We found that the risk score model constructed with five lncRNAs was applicable to multiple GC datasets. We calculated the Pearson correlation coefficient between mRNA and five-lncRNA signature to explore the physiological mechanisms influenced by these biomarkers. Functional analysis suggested that these five lncRNAs play an important role in the initiation and progression of GC through association with known BPs, and further experimental verification of these five prognostic lncRNAs is necessary to understand their function in GC.

Furthermore, we evaluated the prognostic performance of the five-lncRNA signature in several thoracic cancers, including BRCA, ESCA, LUAD, LUSC, and THYM. Since the median can be used as a threshold to arbitrarily divide patients with similar gene expression levels, we used the minimum *p*-value method to obtain the best cut-off point for continuous gene expression (Mizuno et al., 2009). Using this method, we divided the patients with thoracic cancers into different groups according to the minimum *p*-value and found that the five-lncRNA signature could divide patients’ OS of BRCA, LUSC, and THYM into different risk groups. To provide a global view of the prognostic efficiency of the five-lncRNA signature in cancers as much as possible, we performed survival analysis in 27 other types of cancers in TCGA. Although the five-lncRNA signature was not a universal marker for all cancers, the results of Cox regression analysis (Supplementary Figure 1) and Kaplan-Meier survival curves (Supplementary Figure 2) revealed the potential prognostic roles in some other cancers.

## Conclusion

To summarize, this study offers a risk score model according to research and demonstrate in multiple sets of data. Although we only considered lncRNA expression changes and simple clinical information, the prediction model is reliable after multiple verifications. In addition, the identification of lncRNA biomarkers may bring new insights into the potential molecular basis of cancers.

## Data Availability Statement

The original contributions presented in the study are included in the article/Supplementary Material, further inquiries can be directed to the corresponding author/s.

## Author Contributions

JS, WL, and YQ conceived and designed the experiments. JZ, WX, and DW analyzed the data. SL and JZ collected the data. JS and WL validated the method and data. JS and YQ wrote this manuscript. All authors read and approved the final manuscript.

## Conflict of Interest

The authors declare that the research was conducted in the absence of any commercial or financial relationships that could be construed as a potential conflict of interest.

## Footnotes

1 https://www.ncbi.nlm.nih.gov/geo/

2 https://portal.gdc.cancer.gov/
