# Supplementary material for: A Panel of Five-lncRNA Signature as a Potential Biomarker for Predicting Survival in Gastric and Thoracic Cancers
Source: Front Genet. 2021 Apr 13;12:666155. doi: 10.3389/fgene.2021.666155 (PMC8076896; doi:10.3389/fgene.2021.666155)
Supplement: Supplementary file 1 [file Data_Sheet_1.docx]

Supplementary Figures


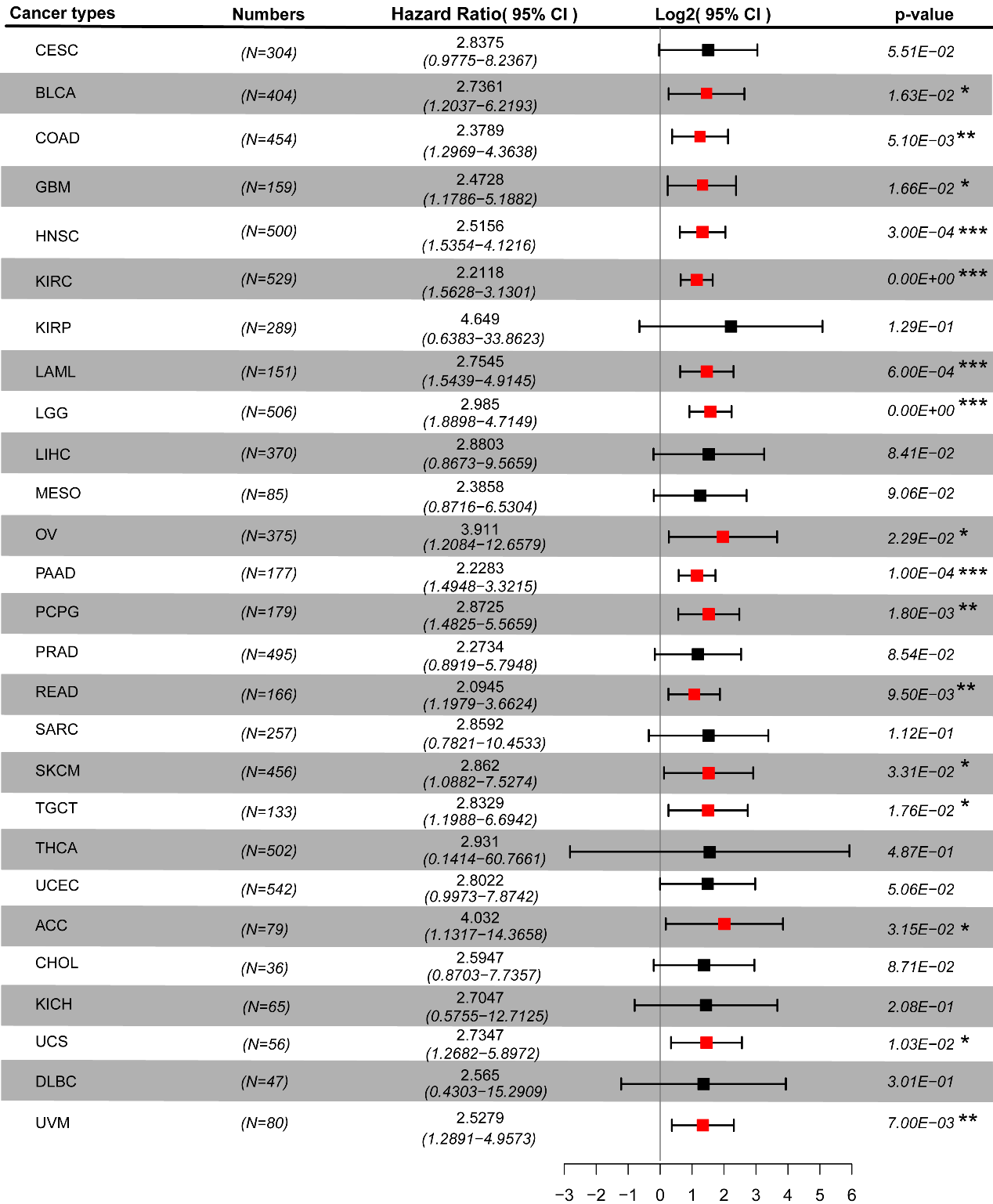


**Figure S1**. Cox regression results of the five-lncRNAs signature in 27 types of cancers from TCGA.

**
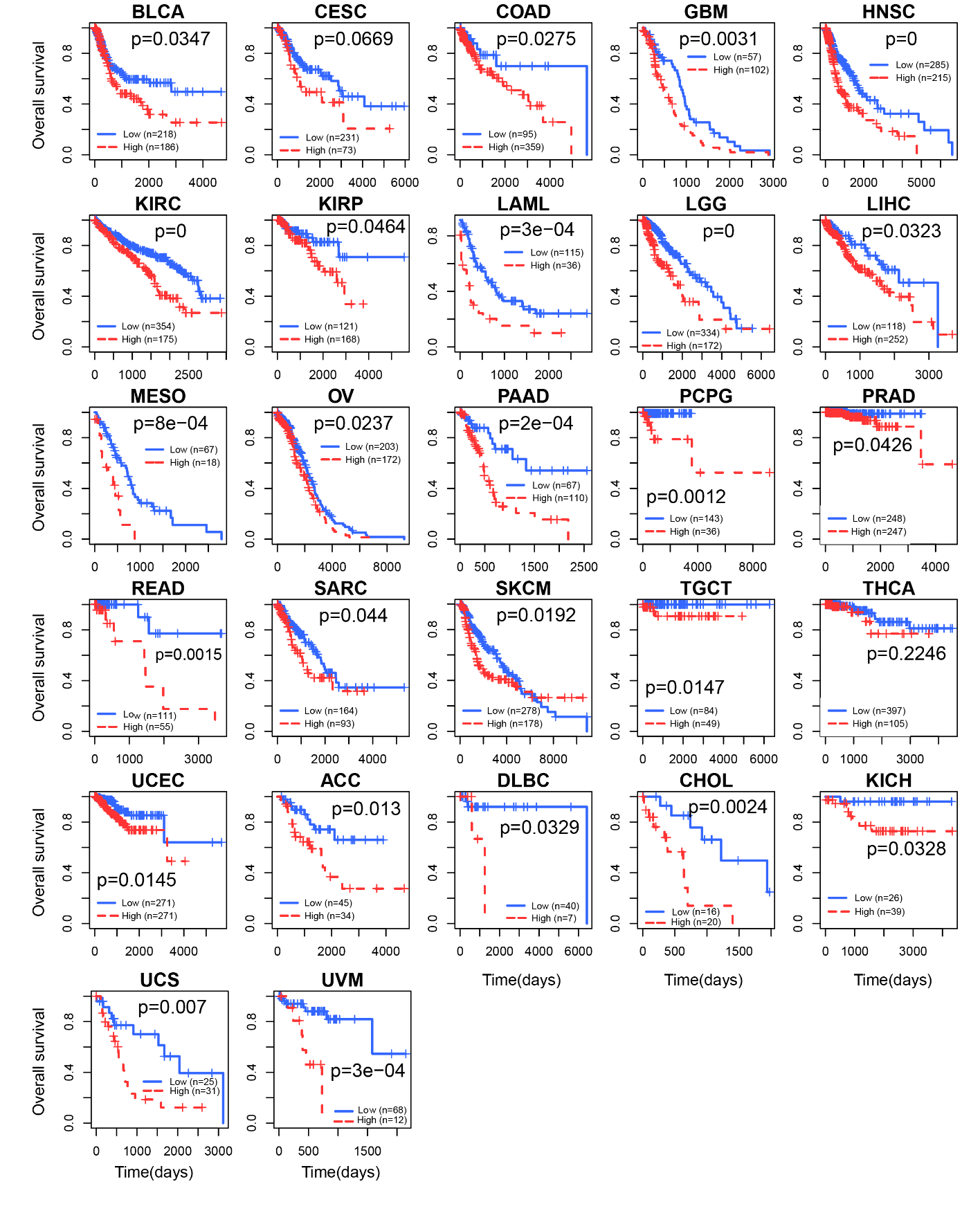
**

**F Figure S2**. Kaplan-Meier curves of the five-lncRNAs signature in 27 types of cancers from TCGA.

Supplementary Table

**Table S1: Description of microarray profiles in gastric cancer**

| **GEO Series** | **Carcinoma sample** | **Tissues** | **Platforms** | **Citation (PMID)** |
| --- | --- | --- | --- | --- |
| GSE62254 | 300 | Gastric | Affymetrix; GPL570 | 25894828, 29725014 |
| GSE15459 | 192 | Gastric | Affymetrix; GPL570 | 19798449, 21471434, 25053715 etc. |
